# Image-Guided Adaptive Cryotherapy for Prostate Cancer Treatment

**DOI:** 10.1007/s10439-025-03833-9

**Published:** 2025-08-29

**Authors:** Eva Beek, Nobuhiko Hata, Kemal Tuncali, Pedro Moreira

**Affiliations:** 1 Department of Radiology, Brigham & Women’s Hospital, Harvard Medical School, 75 Francis St., Boston, 02115 MA USA; 2 Department of Biomedical Engineering, Eindhoven University of Technology, Eindhoven, Netherlands

**Keywords:** Focal cryoablation, MRI-guided cryoablation, Prostate cancer, Adaptive needle placement, 3D Slicer

## Abstract

**Purpose:**

Focal cryoablation is an effective treatment for localized and recurrent prostate cancer, offering reduced risks of side effects. However, treatment success depends on physician experience, as intraprocedural adjustments are required due to needle deflection. To determine whether an image-guided adaptive treatment strategy could reduce the required years of experience, we developed Image-guided Adaptive Cryotherapy (ImAC). We hypothesize that ImAC can be successfully implemented in MRI-guided cryotherapy to optimize needle placement. To test this hypothesis, we conducted a retrospective study comparing the performance of an experienced physician to that of ImAC.

**Methods:**

ImAC was designed to calculate the best subsequent needle location while accounting for needle deflection and iceball formation. After development, its performance was evaluated by simulating 21 needle insertions.

**Results:**

We found that ImAC achieved a higher median minimum ablation margin than the physician (5.0 vs 3.4 mm) while maintaining similar needle placement adjustments (± 7.5 mm).

**Conclusion:**

These results suggest that ImAC has the potential to optimize needle placement and may reduce the years of experience required for effective cryoablation.

## Introduction

Prostate cancer is the second most common cancer worldwide and the fifth leading cause of cancer-related deaths among men as of 2022 [1]. Early detection of prostate cancer has improved due to multiparametric magnetic resonance imaging (mpMRI) and the adoption of prostate imaging reporting and data systems [2]. This advancement is leading to a shift in treatment approach for localized (recurrent) prostate cancer from radical treatments to focal ablation [3, 4].

There are several image-guided focal ablation techniques, such as high-intensity focused ultrasound (HIFU), focal laser ablation (FLA), irreversible electroporation (IRE), and focal cryoablation (FC) [5]. Recent clinical studies indicate that FC is effective for post-radiation recurrent patients and for those with low to intermediate risk, preserving their quality of life by reducing side effects such as incontinence and erectile dysfunction [5–8].

Focal cryoablation is typically guided by either ultrasound (US) or MRI, both of which assist in needle placement and monitoring of tissue freezing. However, the advantages of the technique are fully realized during in-bore MR-guided focal cryoablation, which allows real-time confirmation of the target location. This ensures accurate cryo-needle placement, even in the presence of prostate deformation or patient movement. In settings where in-bore MR-guided procedures are not available, MR–US fusion techniques can aid in tumor localization. However, these computer-assisted fusion approaches are subject to uncertainties in image registration accuracy. MR imaging plays a central role in focal therapy, as the presence of an MR-visible lesion is often a key inclusion criterion for patient selection in focal therapy for prostate cancer.

However, the success of FC treatment depends on precise needle placement to generate a sufficient freezing volume that fully covers the tumor with an appropriate ablation margin [9–11]. Although no official consensus exists on the minimum ablation margin, a 5-mm margin is generally considered the most acceptable guideline. The ablation margin is important because the precise temperature at which cell death occurs, as well as its distribution within the visible iceball, remains uncertain. Additionally, the MRI-based tumor volume tends to underestimate histopathological volumes [12, 13]. Furthermore, FC is performed transperineally, and cryoablation needles are susceptible to deviation from the intended path due to traversing multiple tissues with different mechanical properties over a long insertion distance [14–16], which can affect the placement accuracy. As a result, physicians often need to perform multiple insertions to achieve the desired needle placement [17]. The accuracy of needle placement relies significantly on the physician’s expertise, as they need to use their own expertise to re-plan, estimate the needle deflection, and adjust the insertion accordingly [18, 19].

In brachytherapy, the reliance on physician expertise has been successfully reduced by implementing adaptive treatments, incorporating intraprocedural quality assurance assessment or a treatment planning system [20, 21]. During these treatments, the procedural plan is computationally modified—without relying on physicians’ expertise—by recalculating dose distributions and adjusting needle placements based on the already placed seeds [20–24].

Although such adaptive therapies have been shown to be effective and have been established as image-adaptive brachytherapy, it remains unclear whether they can be integrated into MRI-guided FC. Therefore, we hypothesize that an adaptive treatment strategy can be successfully implemented in MRI-guided cryotherapy to improve ablation outcomes by optimizing subsequent needle placements based on previous insertions and ablation results. We have named this new paradigm “Image-guided Adaptive Cryotherapy” (ImAC). To test this hypothesis, we conducted a retrospective study comparing an experienced physician’s performance to that of ImAC by assessing ablation margin and needle placement similarity.

## Materials and Methods

### Study Design

To determine whether the proposed ImAC performed similarly to the physician, we studied 13 FC patients, involving a total of 21 consecutive needle insertions. During procedures, needle intended locations, tumor segmentation, and all acquired MRI images were saved without disturbing or influencing physician decision-making. This collected information was used to replicate the procedure but using ImAC. The needle locations and the generated iceballs were determined for the original and replicated procedures.

**Fig. 1:**
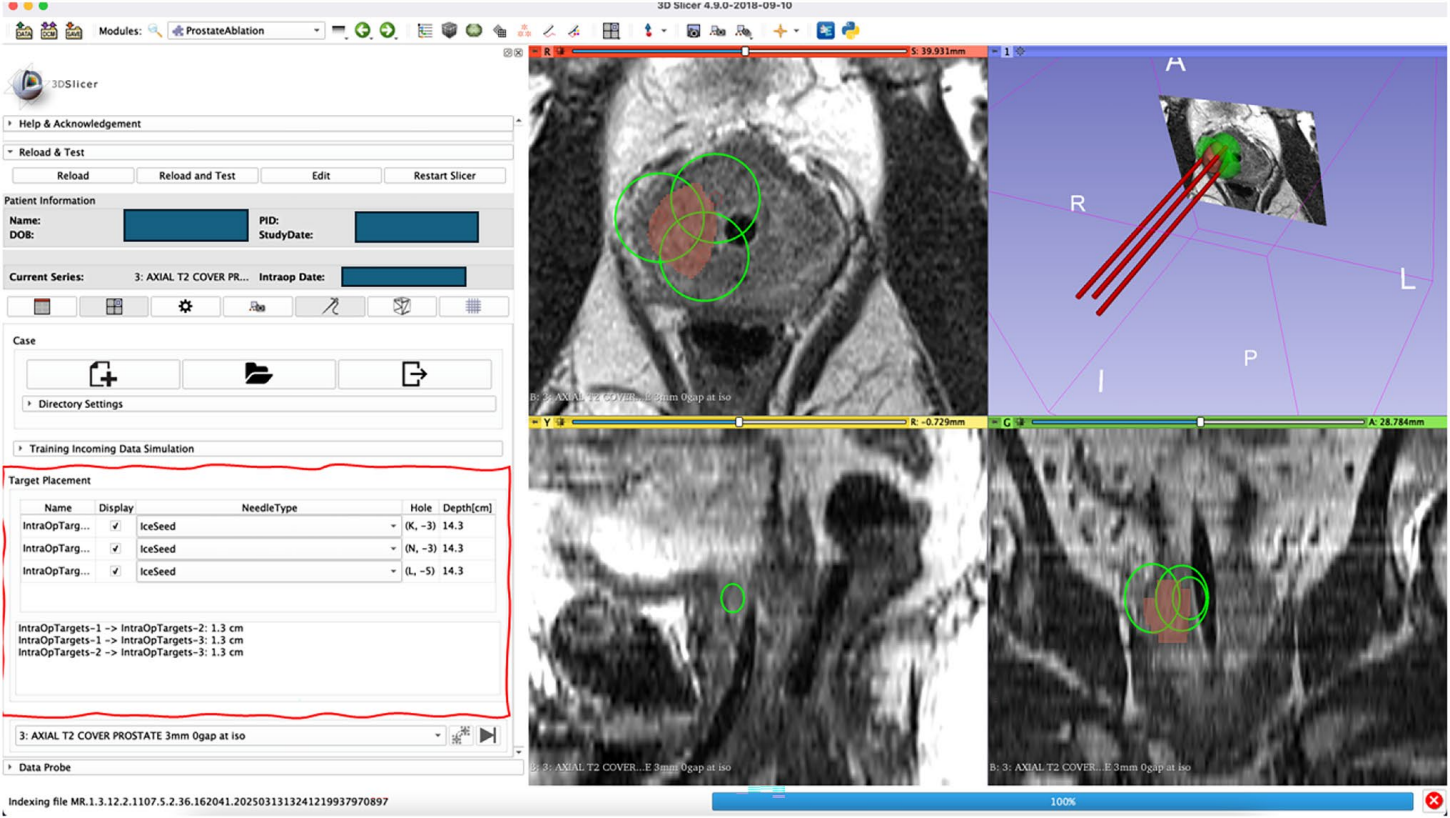
The custom-developed 3D Slicer module for MRI-guided guidance. The physician can define the desired needle placement and the software calculates the template coordinates for each needle to be inserted. The information is provided to the physician on the red-marked area

### Clinical Data Acquisition

We used retrospective data collected from 13 FC procedures performed in a 3-Tesla MRI scanner (MAGNETOM Verio, Siemens Healthineers, Erlangen, Germany) at our institution, Brigham and Women’s Hospital, using an MRI-compatible cryoablation system (SeedNet MRI, Boston Scientific Natack, MA) in the Advanced Multi-modality Image-Guided Operating (AMIGO) suite. This retrospective study was approved by the Institutional Review Board of Brigham and Women’s Hospital. Of these procedures, five used two cryoablation needles, and six used three (Ice Seed, Boston Scientific, Natick, MA). Needle insertion was performed transperineally with the assistance of a needle-guiding template, by one physician with 25+ years of experience in tumor ablation.

### Conventional MRI-Guided Prostate Cryotherapy

The physician utilizes mpMRI to locate the target and define the treatment plan. A template-based approach, similar to the one described by Herz et al. [25], guides the insertion of cryo-needles into the desired locations. After each insertion, an MRI scan verifies needle positioning. This process is repeated iteratively, often requiring multiple insertions until the desired placement is achieved. Once the physician is satisfied with the needle placement, the freezing protocol starts (two freezing cycles of 15 min separated by a 5-minute active thaw), and the iceball growth is continuously monitored using intraprocedural MRI.

At our institution we currently employ a custom-developed 3D Slicer module for MRI-guided guidance (Fig. 1). The integration of ImAC into our guidance workflow was straightforward, as both tools are built on the 3D Slicer platform. In the current workflow, the physician manually determines the desired needle tip placement based on personal experience and a vendor-provided reference chart. The custom-developed 3D Slicer module then applies basic geometric calculations to determine a straight trajectory between the selected target and the nearest available template hole.

**Fig. 2:**
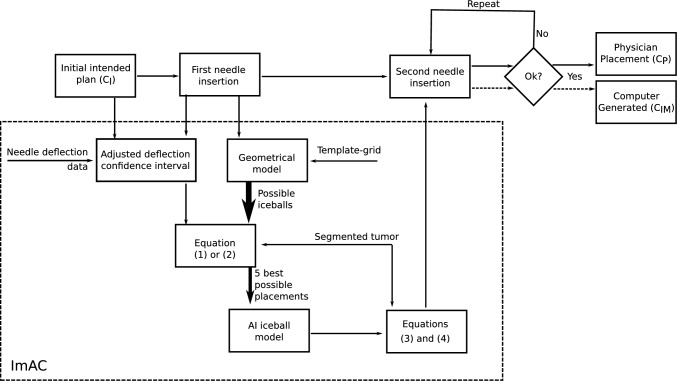
Diagram showing the general workflow of a focal cryoablation procedure, and the procedure with ImAC incorporated in the striped box. First, the physician makes an initial treatment plan (coordinates $C_{\textit{I}}$). During the procedure the plan could be adjusted based on the physician’s decision, resulting in the final physician placement just before freezing (coordinates $C_{\textit{P}}$). Incorporating ImAC reduces the number of adjustments by giving a computer-generated treatment plan (coordinates $C_{\textit{IM}}$). This treatment plan is achieved in multiple steps, considering all possible subsequent needle locations, using a fast geometrical model and a more precise AI iceball model

### Workflow in Image-Guided Adaptive Cryotherapy

The ImAC workflow was designed to seamlessly integrate with current clinical practices, minimizing modifications while enhancing procedural efficiency. Our approach further improves the current workflow incorporating tumor volume, needle deflection, and artificial intelligence-driven (AI) iceball estimation to refine needle placement following the initial insertion. Our method should reduce the number of reinsertions by helping the physician decide on the subsequent placement of the needle (Fig. 2). The method consists of a few steps:

1. **Initial setup:** The procedure begins with manual segmentation of the tumor, registering the needle template, and defining the initial needle targets ($C_{\textit{I}}$). These steps are part of the routine procedure and can be performed in 3D Slicer using an existing module developed by Moreira et al. [26].
2. **First needle annotation:** After the first needle insertion, the clinician annotates the needle tip on the MRI image. The needle produces a large artifact that is clearly visible in MRI images, and the tip is assumed to be located at the center of the distal end of this artifact, as suggested by Mehrtash et al. [27] and performed by several previous studies [16, 28].
3. **Second needle calculation:** Based on the outputs from the previous steps, ImAC calculates the optimal location for the second needle. This calculation accounts for the magnitude of prior deflection and utilizes both geometrical- and AI-based iceball prediction models. Additionally, ImAC determines whether the depth of the first needle should be adjusted.
4. **Second needle annotation** (for cases involving three needles): After the second needle insertion, the clinician annotates the needle tip on the MRI image following the same approach as in the first needle annotation (step 2).
5. **Third needle calculation:** ImAC then calculates the optimal placement for the third needle, incorporating all prior outputs, including the second needle insertion, the magnitudes of previous deflections, and the combined geometrical and AI iceball prediction model.

To implement this workflow, ImAC utilizes iceball models, a retrospective needle deflection dataset, and an optimization algorithm, each described in the following subsections.

#### Retrospective Dataset of Needle Deflection

Previously collected data were analyzed to quantify needle deflection in transperineal insertions. Deflection vectors were calculated as the 3D displacement between the planned and annotated needle tip coordinates in image space. These vectors were used to estimate a multivariate Gaussian distribution, from which the 95% confidence ellipsoid was derived based on the covariance matrix. Principal axes of the ellipsoid were determined using eigenvalue decomposition. This 95% confidence ellipsoid reflects the spatial uncertainty about needle placement and was incorporated into the optimization of subsequent needle location calculations (steps 3 and 5).

The data set consisted of 234 insertions performed in transperineal prostate biopsies. Insertions with deflection exceeding 15 mm were excluded from the database to keep it relevant to cryoablation, where needle deflection is usually less than prostate biopsies [11]. Nevertheless, this dataset allows us to define the trends in needle deflection during transperineal insertions. After filtering, 205 insertions remained in the data set. The 95% confidence interval of the filtered deflections produced a confidence ellipsoid with principal axes of 15.04, 12.82, and 1.96 mm.

#### Geometrical Iceball Model

**Fig. 3:**
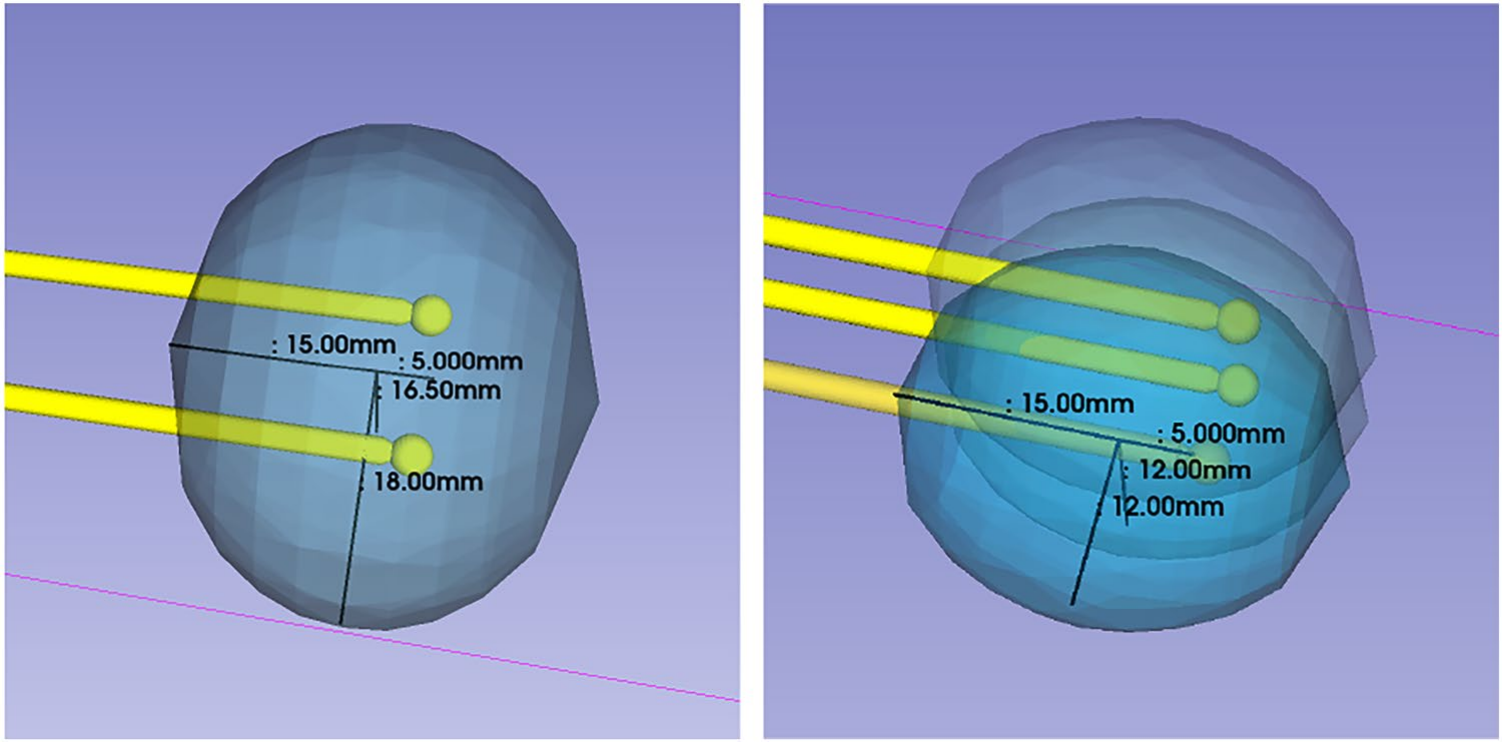
Visualization of the geometrical models used in needle planning. A model consisting of a single ellipsoid is used in two-needle cases (left). A model with three ellipsoids, each centered around a needle used in three-needle cases. The dimensions of one ellipsoid are explicitly annotated, while the other two are shown with reduced opacity (right)

A geometrical model was used to obtain a fast approximation of the formed iceball. This model only uses the positions of the needles to determine the location of the formed iceball and uses the geometry of the $0^\circ$C isotherms as described in the Boston Scientific cryoablation treatment planning guide [29] for the use of IceSeed^™^ 1.5 Straight Needle (Fig. 3). Isotherms represented in this guideline were conducted in a laboratory setting in 37°C temperature-controlled gel. Isotherm measurements were made following two 10-minute freeze cycles separated by a 5-minute passive thaw on each needle type and size. Despite its limitations in accuracy, the geometrical model provides a simple but effective approach to the initial optimization step without requiring significant computational power.

#### AI-Based Iceball Model

Different from the geometrical model, the AI-based model considers the heat-sink effect from the urethral warmer catheter and is able to capture the synergistic effect between more than one cryo-needle. The model was validated in our previous study [30]. The model is based on the 3D U-Net architecture, trained on retrospective data from 38 focal cryoablation procedures. The model takes as input the MR image acquired after needle placement, with the distal 15 mm of each needle marked using a constant pixel value outside the normal image intensity range. The model outputs a label map representing the predicted isotherm boundaries. The model was trained using the Adam optimizer with an experimentally defined learning rate of $10^{-3}$, a batch size of 2, for 400 epochs, and optimized with a Dice loss function. The technical implementation has been described previously [30]. The model achieved a mean Dice Similarity Coefficient (DSC) of 0.79 and an average Hausdorff distance of 1.8mm. Despite the relatively small training dataset, this level of accuracy has been considered acceptable for ablation planning purposes [31].

#### Needle Placement Optimization

After the physician defines the initial plan and inserts the first needle, ImAC optimizes the placement of subsequent needles. The subsequent needle location is determined by evaluating all possible placement locations considering the grid-template restrictions and clinical guidelines on the distance between needles. The possible locations are between 1 and 2 cm from the first needle, following the guidelines from Boston Scientific cryoablation treatment planning guide [29] and a study by Taimur et al. [32]. These possible placement locations are defined at seven different depths, positioned 5, 10, and 15 mm above and below the depth of the first needle. To reflect the correct location at different depths, the orientation of the needle template is taken into account.

For each possible location, the geometrical model and the 95% confidence ellipsoid are jointly applied to take into account iceball size and needle deflection at the same time. The geometrical model is used to have a fast approximation of the iceball shape only based on the needle locations (Sect. 2.4.2). Furthermore, to maximize the likelihood of correct needle placement within the target area, we use the 95% needle placement confidence interval ellipsoid to take into account potential deflections (Sect. 2.4.1). The ellipsoid is centered around the possible placement location, with its principal axes aligned to the direction of the placement error of the first needle insertion. The deflection direction is determined by calculating the difference between the initially planned needle tip position and the actual achieved position in the first insertion, obtained from Steps 1 and 2, respectively. The overlap between the tumor segmentation and the iceball generated by the geometrical model, as well as the overlap between the tumor segmentation and the 95% confidence interval ellipsoid, are calculated and combined using Eqs. 1 and 2 for two- and three-needle cases, respectively. In these equations,

- $V_{\text {Geo}}$ represents the iceball volume calculated by the geometrical model.
- $V_{\text {T}}$ represents the volume of the tumor segmentation.
- $V_{\text {C, Nn}}$ represents the volume of the 95% confidence interval ellipsoid centered around needle two ($\text {N}_2$) or three ($\text {N}_3$).
- $W_1$ and $W_2$ are weights, both set to 0.5 to reflect the equal importance of tumor coverage and uncertainty of placement.

1 $$\begin{aligned} O_{\text {S2}}= & (V_{\text {Geo}} \cap V_{\text {T}}) \cdot W_1 + (V_{\text {C, N2}} \cap V_{\text {T}}) \cdot W_2 \end{aligned},$$

2 $$\begin{aligned} O_{\text {S3}}= & (V_{\text {Geo}} \cap V_{\text {T}}) \cdot W_1 + \nonumber \\ & \frac{(V_{\text {C, N2}} \cap V_{\text {T}}) + (V_{\text {C, N3}} \cap V_{\text {T}})}{2} \cdot W_2 \end{aligned}.$$

The five locations with the highest overlap score ($O_{\text {S}}$) were further evaluated by generating their corresponding iceball with the AI model (Sect. 2.4.3). This iceball was evaluated, by calculating the overlap score between the iceball and the tumor segmentation and by calculating the ablation margin (M) with Eq. 3. Combining the two resulted in a performance score ($P_{\text {S}}$) with Eq. 4.

In these equations,

- $V_{\text {AI}}$ represents the iceball volume calculated by the AI model.
- $V_{\text {T}}$ represents the volume of the segmented tumor.
- $\partial V_{\text {AI}}$ and $\partial V_{\text {T}}$ denote the boundaries of the AI iceball model and tumor segmentation, respectively.
- $d(p, q)$ represents the Euclidean distance between the points $p$ and $q$.
- $W_1$ and $W_2$ are weights that can be adjusted depending on the physician’s preference. In this study, they are both set to 0.5 to reflect the equal importance of tumor coverage and ablation margin.

3 $$\begin{aligned} M= & \min \{ d(p, q) \,|\, p \in \partial V_{\text {AI}}, q \in \partial V_{\text {T}} \} \end{aligned},$$

4 $$\begin{aligned} P_{\text {S}}= & (V_{\text {AI}} \cap V_{\text {T}}) \cdot W_1 + M \cdot W_2 \end{aligned}.$$

The location with the highest performance score was recommended to the physician. If two locations had the same score, both were suggested and it is up to the physician to decide which one to use. Additionally, an optimal depth for the first needle was provided, enabling the physician to make adjustments as needed without requiring reinsertion.

### Validation Study

A retrospective study was performed to evaluate the performance of ImAC. The procedure was simulated by incorporating ImAC and following the specific steps mentioned in paragraph 2.4. The results were compared to the physician.

We evaluated the results by measuring the difference in needle location on the axial plane between the physician’s initial intended planned targets ($C_{\textit{I}}$), physician’s final placement ($C_{\textit{P}}$), and ImAC’s suggestion ($C_{\textit{IM}}$).

Furthermore, we compared the generated iceballs by calculating the minimum ablation margin and percentage of margin above 5 mm. Data were summarized as median + IQR. Since the distance and margin distributions were not normally distributed as indicated by histogram and Shapiro–Wilk test ($p > 0.05$). We then used a Wilcoxon signed-rank test, to determine which means were significantly different from our program. We considered differences significant at $p < 0.05$.

## Results

**Fig. 4:**
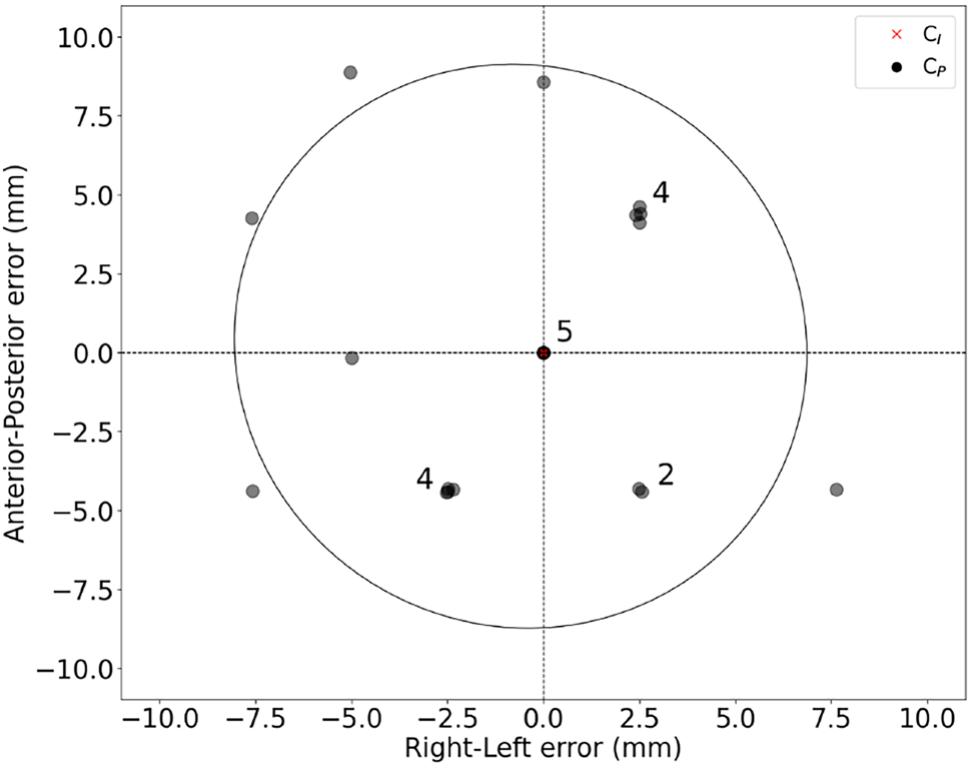
Deviation from the intended planned placement (0,0) ($C_{\textit{I}}$) by the physician ($C_{\textit{P}}$) on the axial plane and its 95% confidence ellipsoid

**Fig. 5:**
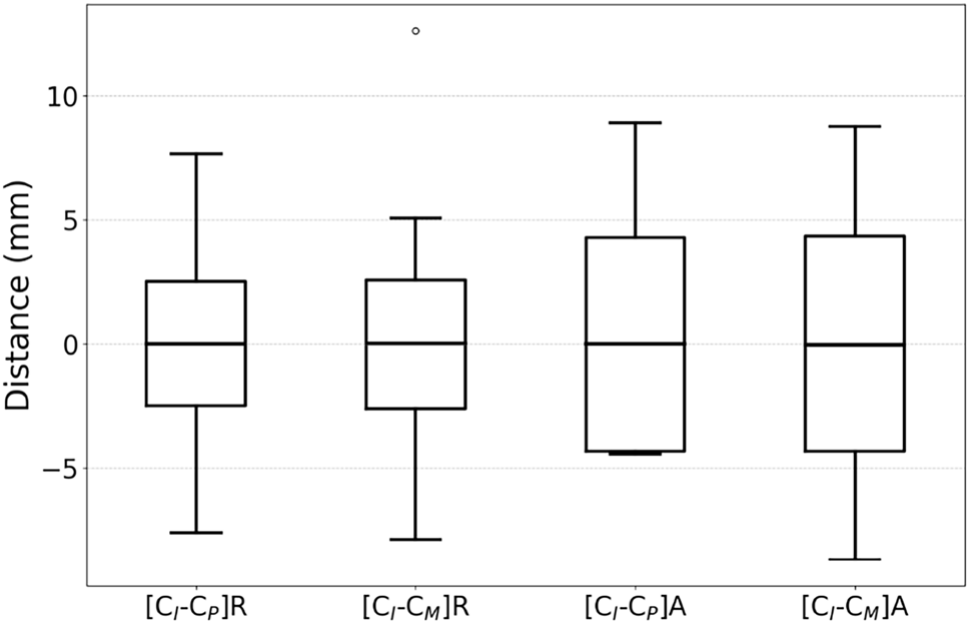
Deviation from intended planned placement ($C_{\textit{I}}$) by the physician ($C_{\textit{P}}$) and ImAC ($C_{\textit{IM}}$) on axial plane in right–left (R) and anterior–posterior (A) direction. Everything was not statistically different ($p<0.05$)

Our retrospective study consisted of 21 needle placement optimizations, of which 13 were second and 8 were third needle placements. During the case, the physician often adapted the initial plan ($C_I$) and used different grid holes to compensate for needle deviation. In this study, such adaptations occurred in 16 of 21 needle insertions, with adaptations of ± 7.5 mm from the intended plan in the axial plane (Fig. 4). It should be noted that ImAC suggested adjustments of similar magnitude compared to the adjustments made by the physician (Fig. 5).

**Fig. 6:**
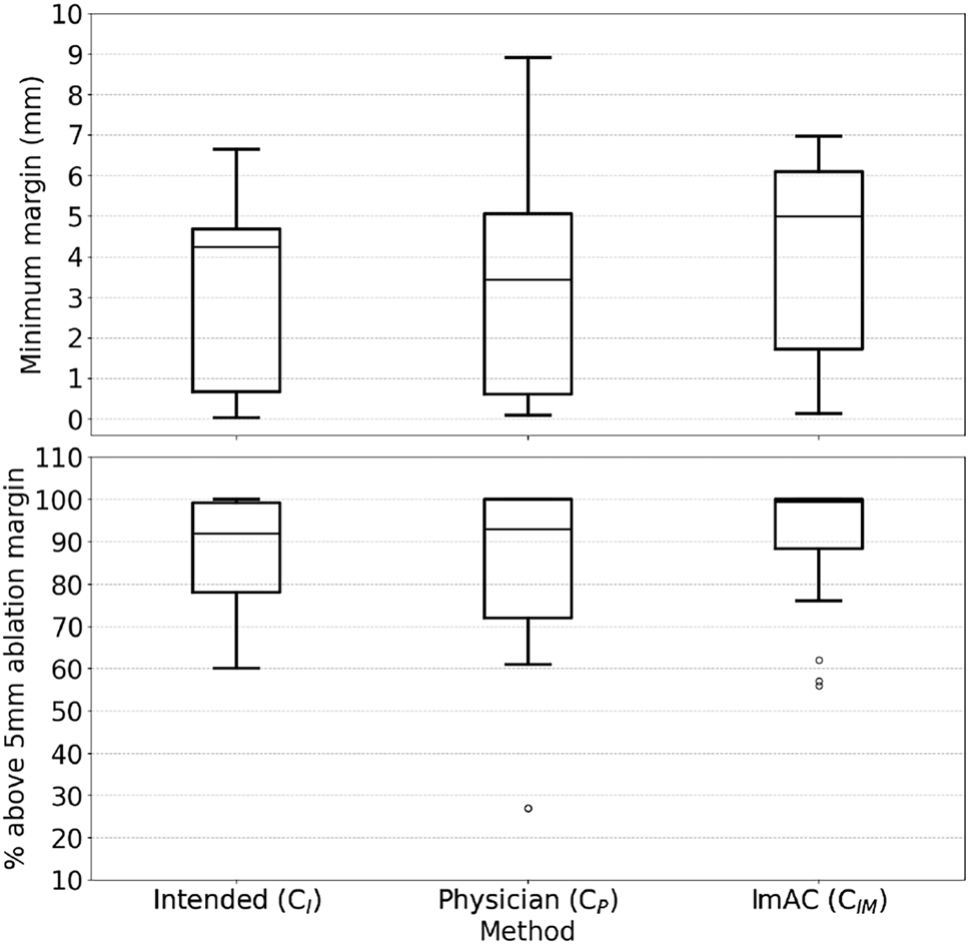
Effect of needle placement method on minimum ablation margin (top) and percentage of ablation margin above 5 mm (bottom). With * indicating statistical difference ($p<0.05$) between the two methods indicated by the brackets

On average, the needle placements suggested by ImAC achieved higher ablation margins, as reflected in the higher minimum ablation margin and percentage of ablation margin above 5 mm (Fig. 6). The Wilcoxon signed-rank test showed no significant differences between the physician and ImAC for the minimum ablation margin ($p=0.13$) and percentage above 5-mm margin ($p=0.22$) (Table 1). Still, ImAC’s negative skewness in the minimum ablation margin suggests a stronger concentration of higher minimum ablation margins compared to the physician. This suggests that, while statistical significance may not fully capture the difference, ImAC is likely to achieve higher ablation margins more consistently than physician placement.

Incorporating ImAC into the normal procedure workflow would take 19.96 s (IQR [19.51, 35.85]) of computational time per needle insertion. Although this time does not include the initial setup and needle annotations that are user dependent, it is well within an acceptable range to be used intraprocedural.

**Table 1:** Effect of needle placement method on ablation margin and needle deviation in cryoablation

Measurement	Intended ($C_{\textit{I}}$)	Physician ($C_{\textit{P}}$)	ImAC ($C_{\textit{IM}}$)
Minimum ablation margin (mm)	4.22 [0.6, 4.6]	3.43 [0.6, 5.0]	5.00 [1.7, 6.1]
Percentage above 5-mm ablation margin (%)	92.0 [78.0, 99.1]	93.0 [72.0, 100.0]	99.5 [88.0, 100.0]
Deviation R-direction (mm)	–	0.00 [$-$2.5, 2.5]	0.01 [$-$2.6, 2.6]
Deviation A-direction (mm)	–	0.00 [$-$4.3, 4.3]	$-$0.01 [$-$4.3, 4.3]

**Fig. 7:**
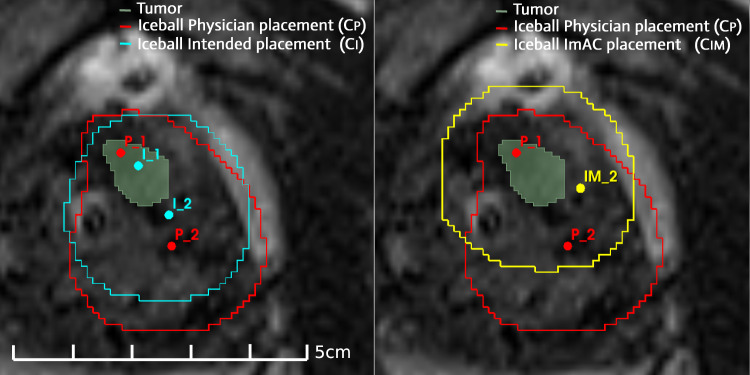
Case A, effect of needle placement method on needle location and iceball formation. Normal procedure where the physician ($C_{\textit{P}\_\textit{2}}$) deviated from the intended plan ($C_{\textit{I}\_\textit{2}}$) (left). Incorporating ImAC ($C_{\textit{IM}\_\textit{2}}$) gains a more evenly distributed ablation margin than the physician (right). Both ($C_{\textit{P}\_\textit{2}}$) and ($C_{\textit{IM}\_\textit{2}}$) are based on the first needle placement ($C_{\textit{P}\_\textit{1}}$)

**Fig. 8:**
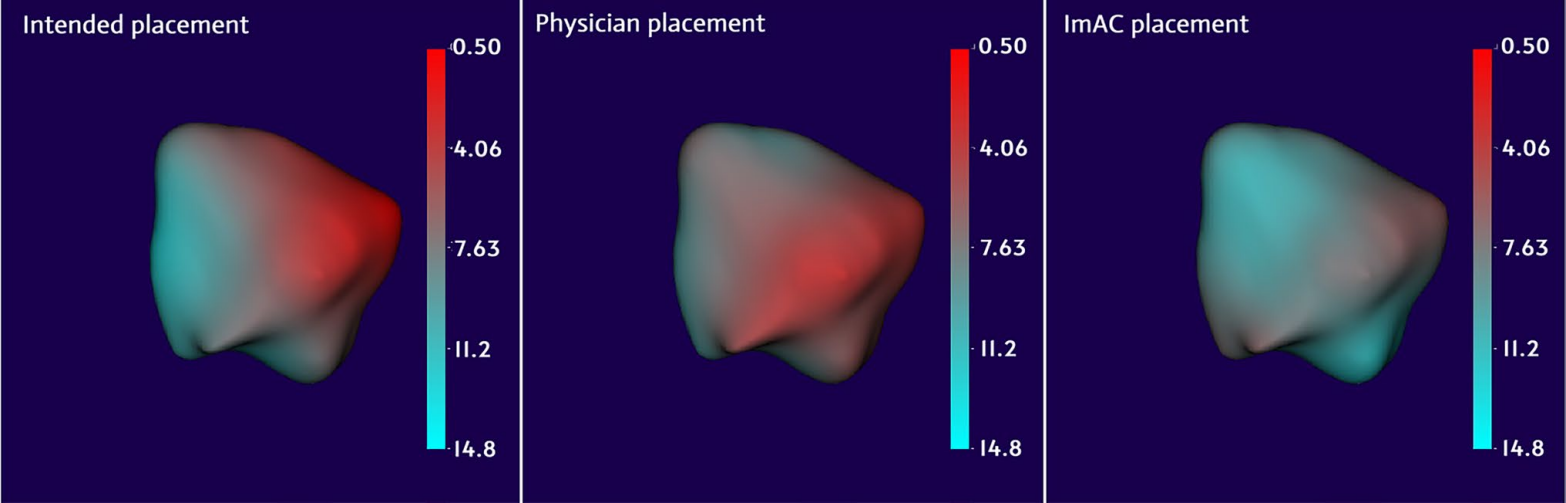
Case A, heatmap showing effect of needle placement method (intended planned placement, physician and ImAC) on ablation margin. Blue corresponds to the parts with a high ablation margin, and red corresponds to a low margin

Figures 7 and 8 show a representative case in which ImAC produced an outcome with larger ablation margins than the placement adjusted by the physician and the initial intended placement. Both the physician and ImAC adjusted the intended plan based on the first needle placement to compensate for needle deflection and optimize the treatment result (Fig. 7). Pairwise statistical analysis between all placements of the physician and ImAC revealed no significant differences in the right–left direction ($p = 0.42$) nor in the anterior–posterior direction ($p = 0.58$) (Table 1). Suggesting that the physician and ImAC make adjustments of the same reasonable magnitude. However, in certain cases, the adjustment occurred in a different and more favorable direction resulting in better iceball margins (Fig. 7) suggesting that there could have been a better placement.

## Discussion

The study presented and evaluated the use of a rapid computer-generated adaptive needle planning tool to optimize needle placement during image-guided cryoablation. The results demonstrated that ImAC was fast, 19.96 s (IQR [19.51, 35.85]) per insertion, while gaining higher ablation margins than the physician and similar deviations from the intended plan as the physician. The results also demonstrated that ImAC achieved a minimum ablation margin of 5.0 mm (IQR [3.3, 6.2]), meeting the threshold suggested by multiple studies, which recommend a minimum margin of at least 5 mm for effective focal prostate cryoablation [9, 12].

Moreover, Fig. 6 shows that ImAC produced less variability in the amount of tumor covered with an ablation margin above 5 mm. This observation may indicate a potential benefit of intraoperative planning software, even though the statistical comparison did not reach significance. In at least 50% of the cases, the complete tumor was covered with an ablation margin above 5 mm, whereas the physician achieved this in less than 25% of the cases, indicating that ImAC produced more consistent results. These percentages are lower than Overduin et al. who reported 72%. However, differences in measurement methods may explain some variations. ImAC employs a surface-based distance calculation, capturing the full 3D structure of the ablation zone, whereas Overduin et al. measured margins in only three linear directions. This methodological difference likely accounts for the seemingly lower margins reported for ImAC, as its approach imposes a stricter, more comprehensive assessment of ablation coverage. This is also reflected in the higher minimum ablation margin of 7.5 mm reported by Overduin et al.

The differences between the planned and final needle placements were expected, as needle deviations are common and often necessitate replanning and reinsertion during the procedure [11, 17]. The adjustments observed by both the physician and ImAC were of a similar magnitude, with a 95% confidence ellipsoid in the axial plane of approximately ± 7.5 mm. This value correlates with the findings from Moreira et al., who reported in-plane placement errors of 6.5 ± 1.8 mm [26]. These results suggest that ImAC could perform at a level comparable to experienced physicians with over 25 years of expertise, potentially reducing the learning curve for effective cryoablation while maintaining treatment quality.

While the results are encouraging, the study has a few limitations. First, it included only 21 needle insertions, all performed by a single physician and analyzed retrospectively, which may induce bias. In addition, the small effect sizes between groups and the limited statistical power of the Wilcoxon test with a small sample size likely contributed to a high Type II error rate ($\beta$), meaning that the nonsignificant result does not necessarily indicate the absence of an effect, but rather that an effect was not detected. Expanding the dataset to include more cases from multiple physicians would help mitigate this limitation. Second, needle annotations in steps 2 and 4 were performed selecting the center of the needle artifact as performed in previous studies [16, 17, 27]. However, it is well known that needle artifacts are larger than the actual needle and it is subject to variation. Mehrtash et al. reported a median variation between observers of 0.88 mm, which may have a minor impact on our results [27] A possible solution to reduce variability in needle tip annotation is to use AI models to automatically track the needle tip [27, 33]. It is also worth noting, that the prediction model may exhibit a bias toward procedures involving two or three needles, potentially leading to reduced accuracy in cases with four or more needles. Alternative AI-based models for iceball prediction have been proposed and could be explored or integrated in future work to improve generalizability. Lastly, the data used to estimate prior deflections was derived from transperineal biopsies rather than cryoablation needles. Although filtering was applied to mitigate this difference, expanding this dataset may result in better deflection estimations.

This study focused on MRI-guided in-bore focal cryoablation, but ImAC also has the potential to be applied to ultrasound-guided cryotherapy [5]. This could be achieved by fusing ultrasound and preoperative MRI images or by training an AI model specifically for ultrasound data.

In conclusion, we introduced a novel computer-generated adaptive needle planning tool for MRI-guided focal cryoablation of prostate cancer. ImAC has the potential to optimize needle placement and may reduce the years of experience required for effective cryoablation while maintaining treatment quality.

## Data Availability

The software code and final trained model developed in this study are available on GitHub. In addition, the authors can provide access to synthetic MR images and de-identified annotations to enable reproducibility using our software. In accordance with IRB protocol and HIPAA regulations, no patient Protected Health Information (PHI), including MRI images, will be publicly shared.
